# Case-control study of maternal and fetal outcomes in beta thalassaemia trait during pregnancy

**DOI:** 10.1371/journal.pone.0327132

**Published:** 2025-07-17

**Authors:** Shyamali Thilakarathne, Udayanga P. Jayaweera, Sanjaya Uduweralla, Sudath Pathinisekara, Thushari U. Herath, Anuja Premawardhena

**Affiliations:** 1 Department of Medical Laboratory Science, Faculty of Allied Health Sciences, University of Peradeniya, Peradeniya, Sri Lanka; 2 Divisional Hospital, Menikhinna, Sri Lanka; 3 Teaching Hospital, Kurunegala, Sri Lanka; 4 Department of Medicine, Faculty of Medicine, University of Kelaniya, Ragama, Sri Lanka; Al Muthanna University, IRAQ

## Abstract

Individuals with beta thalassaemia trait are not expected to have clinically significant morbidities besides mild anaemia. Pregnancy would exaggerate the anaemia in beta thalassaemia traits, but how this could affect maternal and fetal outcomes is unclear. Previous studies on maternal and fetal outcomes in beta thalassaemia trait have been inconsistent and even contradictory. Thus, we aimed to study the outcomes of pregnancy in pregnant women with beta-thalassemia trait. The prospective case-control study included 120 pregnant women with beta thalassaemia trait and 120 normal pregnant women. Participants in the case and control groups were matched according to maternal age, gestational age and number of previous pregnancies. All participants were followed up at similar intervals for the duration of the pregnancy for haemoglobin variations, transfusion requirements and maternal/fetal problems without interfering with the management. We identified that beta thalassaemia traits did not experience noteworthy symptoms in any of the three trimesters: except for headache. Haemoglobin level, and red cell indices in all three trimesters were significantly lower among cases than controls. In both groups hemoglobin levels dipped in the 2^nd^ trimester, only to rise in the 3^rd^ trimester, to reach values similar to those in the 1^st^ trimester. Individuals with beta thalassaemia trait were transfused more blood during the 2^nd^ and 3^rd^ trimesters based on lower Hb levels of the mother a decision not prompted by any notable maternal or fetal complications. No statistically significant differences were observed for pregnancy complications, perinatal or neonatal outcomes. During labor, the rate of caesarean deliveries was significantly higher among cases with no definite indications for such in most cases.

## Introduction

An estimated 80–90 million people globally amounting to 1.5% of the world population are carriers for beta thalassemia [1]. Half of them live in South & Southeast Asia. In Sri Lanka, the carrier rate for beta thalassaemia is 2.5%, with very similar rates in the three major ethnic groups [2].

Beta-thalassemia traits have a reduced production of the beta globin chain and are associated with a small size of red blood cells (reduced mean corpuscular volume) and decreased hemoglobin level (reduced mean corpuscular hemoglobin) [3]. The mutational spectrum of beta thalassaemia is highly heterogeneous and varies significantly by geographic region and ethnic background [4]. Individuals with beta thalassaemia trait are usually asymptomatic and besides mild anaemia detected on a full blood count in most individuals are clinically indistinguishable from those without the condition.

The pregenancy-associated physiological changes in the blood such as; 30% increase in red blood cell volume or 40% increase in total blood volume could potentially cause problems for beta thalassaemia traits during pregnancy [5]. It is necessary to clinically investigate whether beta thalassaemia traits can tolerate such drastic changes and whether these women have increased rates of adverse pregnancy outcomes as a result. Previous studies on this subject have shown that pregnancies in beta thalassaemia trait women can have a higher risk of adverse obstetric outcomes, such as pre-term birth and low birth weight [6] contrastingly some other studies contradict this [7]. Thus the available literature is ambiguous relating to pregnancy outcomes of beta thalassaemia traits. The current study was designed as a comparative prospective case-control study in an attempt to assess maternal and fetal outcomes in pregnancies with beta thalassaemia trait.

## Materials and methods

### Study design and study population

This prospective case-control study was conducted by recruiting pregnant women attending the antenatal clinics of the Kurunegala Teaching Hospital, the largest tertiary care hospital in the northwestern province of Sri Lanka. This hospital has a bed strength of 1700 and conducts 14,000 deliveries per annum. The study included 120 cases and 120 controls. WinPepi software, version 11.65, was used to calculate sample size at the significance level of 5% and power 80%. Cases were defined as those with confirmed beta thalassaemia trait and controls were those without beta thalassaemia trait on HPLC. Individuals attending clinics of two specialist obstetricians in the same hospital were selected at random. Ethical clearance was obtained from the ethics review committee, Teaching Hospital Kurunegala (Ref. No: THK/HIRU/ERC/20/12) and the University of Kelaniya Ethics Review Committee (Ref. No: P/209/11/2018). Only those who gave written consent and had a minimum of three follow-up visits to the antenatal clinic and who delivered in the selected hospital were recruited. The study started in February 2021 and the sample size was attained in July 2022. Individuals with thalassaemia major/intermediate type, any other inherited blood disorders and severe clinical conditions such as renal disease, heart disease were excluded from the study. In addition, women with a history of infertility, two or more consecutive previous pregnancies resulting in spontaneous abortion or neural tube defects in previous babies were also excluded.

### Data collection and sampling

At the time of recruitment, all pregnant women were interviewed to gather demographic data including name, age, gender, address, and ethnicity. Past medical records were carefully assessed for any information on chronic disease conditions and inherited blood disorders. Physical data, such as the participant’s height and weight, were collected. They were followed up at similar intervals for the duration of the pregnancy and an interviewer-administered questionnaire was filled out for each woman. Clinical information such as Hb level variations, transfusion requirements and maternal/fetal problems of pregnant women were obtained from their medical records without interfering with the management.

Gestational age was calculated from the first day of the last menstrual period. The diagnosis of heterozygous beta thalassaemia was established on the basis of Hb A2 level > 3.5% using HPLC method (Bio-Rad variant II), and the subjects with Hb A2 level below 3.2% were considered normal controls. The BMI of each participant was calculated as weight at the booking visit in kilograms divided by height in square meters. According to the Sri Lankan BMI classification for adults, its range ≥25 kg/m^2^ is considered obese, 23–24.9 kg/m^2^ is considered overweight, 18.5–22.9 kg/m^2^ is considered normal, and<18.5 kg/m^2^ is considered underweight [8].

Poor pregnancy outcomes or complications included gestational diabetes mellitus, pregnancy-induced hypertension, pre-eclampsia, eclampsia, intrauterine growth restriction (IUGR) (when intrauterine growth under 10% by serial sonography especially in 3^rd^ trimester of pregnancy), polyhydramnios (amniotic fluid index more than 24 cm), oligohydramnios (amniotic fluid index 5 cm or less, placental abruption and preterm labor (labor pain under 37 weeks of gestational age). Labor and perinatal outcomes included; cesarean delivery, postpartum hemorrhage, maternal packed-cell transfusions, Apgar score at 5 minutes less than 7, resuscitation at birth, liquor, birth weight, body length, head circumferences, neonatal ICU admission, congenital abnormalities and neonatal complications.

### Statistical analysis

Statistical analyses were performed using SPSS version 21.0 (IBM Corp. Released 2012; IBM SPSS Statistics for Windows, Armonk, NY: IBM Corp). The results of quantitative variables were presented as mean ± standard deviation (SD) and those of categorical variables by frequency (percentage). t-test or Mann–Whitney U-test was used to compare continuous variables, whereas categorical variables were compared using the chi-square test. The association of variables was tested by Pearson’s correlation coefficient. P values of.05 or less were considered statistically significant.

## Results

There were 120 cases and 120 controls recruited for the study. The age of the case-control pairs ranged between 18–42 years. The mean HbA2 level of the case group was 4.69 ± 0.47% while that of the control group was 2.47 ± 0.38%.

The baseline characteristics of subjects in the two study groups are shown in Table 1. There was no statistically significant difference between these groups regarding these baseline characteristics.

**Table 1:** Baseline characteristics of case and control groups.

	Case (N = 120)	Control (N = 120)	P value
Age category			0.675
< 20	6	6	
21–30	66	65	
31–40	47	49	
> 40	1	0	
Ethnicity			0.739
Sinhalese	112	112	
Muslims	5	4	
Tamils	3	4	
Education level			0.650
< Secondary	61	68	
Secondary	50	45	
Diploma	3	1	
Graduated	6	6	
Occupation			0.740
Unemployed	73	68	
Skilled profession	27	32	
Non skilled profession	20	20	
Parity			–
Nulliparity	37	37	
Multiparity	83	83	

The number of women with each clinical feature was high in the case group compared to the control group in almost all trimesters (Table 2). However statistically significant differences were observed only for headaches in the 3^rd^ trimester.

**Table 2:** Significant clinical features and number of blood transfusions in cases and controls in three trimesters.

	End of 1^st^ trimester				End of 2^nd^ trimester				End of 3^rd^ trimester			
	Cases^*^ (N = 120)	Controls^*^ (N = 120)	OR (95% CI)	P value	Cases^*^ (N = 120)	Controls^*^ (N = 120)	OR (95% CI)	P value	Cases^*^ (N = 120)	Controls* (N = 120)	OR (95% CI)	P value
**None**	57(47.5)	64(53.3)	0.8 [0.5, 1.3]	0.425	68(56.7)	85(70.8)	0.5 [0.3, 0.9]	0.050	53(44.2)	78(65)	0.4 [0.3,0.7]	0.004^a^
**Morning sickness**	48(40)	45(37.5)	1.1 [0.7, 1.9]	0.547	14(11.7)	13(10.8)	1.1 [0.5, 2.4]	0.590	11(9.2)	8(6.7)	1.4 [0.6,3.7]	0.462
**Tiredness**	28(23.3)	19(15.8)	1.6 [0.9, 3.1]	0.197	27(22.5)	19(15.8)	1.5 [0.8, 3.0]	0.245	38(31.7)	24(20)	1.9[1.0,3.3]	0.066
**Headache**	16(13.3)	14(11.7)	1.2 [0.5, 2.5]	0.555	16(13.3)	9(7.5)	1.9 [0.8, 4.5]	0.196	21(17.5)	7(5.8)	3.4 [1.4,8.4]	0.011^b^
**Shortness of breath**	14(11.7)	6(5)	2.5 [0.9, 6.8]	0.102	7(5.8)	5(4.2)	1.4 [0.4, 4.6]	0.503	18(15)	13(10.8)	1.5 [0.7,3.1]	0.372
**Chest pain**	2(1.7)	1(0.8)	2.0 [0.2, 22.6]	0.561	1(0.8)	1(0.8)	1.0 [0.1, 16.2]	0.605	5(4.2)	3(2.5)	1.7 [0.4,7.3]	0.463
**No of blood transfusion**	0	0	–	0.316	22(18.3)	0	–	<0.001 ^c^	20 (16.7)	1 (0.83)	23.8[3.1,180.5]	<0.001^c^

**OR**: Odds Ratio; **CI**: Confidence Interval.

* Data expressed as n (%) unless otherwise indicated.

^a^Adjusted P value = 0.038.

^b^Adjusted P value = 0.168.

^c^Adjusted P value = < 0.001.

Haematological parameters (Hb, MCV, MCH, MCHC and RDW) were significantly different between case and control groups as expected (Table 3).

**Fig 1:**
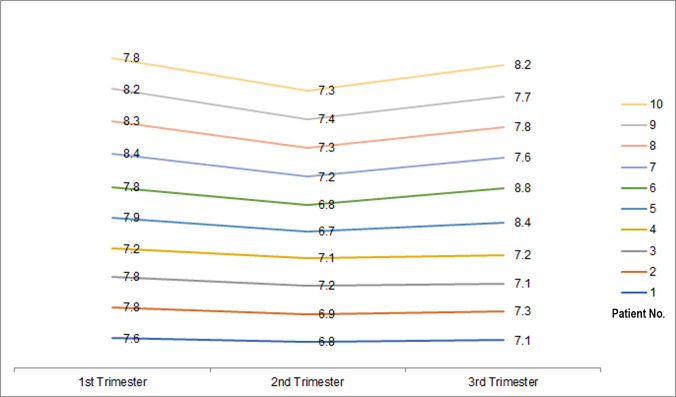
The changes in Hb levels (g/dL) during three trimesters in 10 randomly selected beta thalassaemia trait mothers who had undergone blood transfusions.

The number of blood transfusions received during the pregnancy showed a statistically significant difference between the two groups, with a higher number of cases having received transfusions in the 2^nd^ (n = 22) and 3^rd^ (n = 20) trimesters than the controls. Among the control group, only one pregnant woman had a blood transfusion in the 3^rd^ trimester. In the 1^st^ trimester, none of the cases and controls had a blood transfusion. Fig 1 shows changes in Hb levels during three trimesters in 10 randomly selected beta thalassaemia trait mothers who had undergone blood transfusions.

No participants were observed with preeclampsia, eclampsia and placental abruption in both cases and controls. None of the complications show statistically significant differences between cases and controls (Table 4).

**Table 3:** Haematological parameters of cases and controls in three trimesters.

Parameter	1^st^ trimester					2^nd^ trimester				3^rd^ trimester			
	Cases		Controls		P value	Cases		Controls	P value	Cases		Controls	P value
	N	Mean±SD	N	Mean±SD		N	Mean±SD	Mean±SD		N	Mean±SD	Mean±SD	
**Hb** (g/dL)	118	9.50 ± 0.64	116	11.62 ± 1.15	<0.001	117	9.03 ± 0.85	11.01 ± 1.94	<0.001	117	9.52 ± 0.68	11.74 ± 1.81	<0.001
**MCV** (fL)	111	61.48 ± 3.08	115	81.76 ± 6.92	<0.001	110	62.87 ± 3.00	85.90 ± 23.48	<0.001	114	64.14 ± 3.15	85.90 ± 22.16	<0.001
**MCH** (pg)	111	20.65 ± 1.55	115	27.76 ± 2.45	<0.001	110	21.06 ± 1.51	28.5 ± 7.95	<0.001	114	21.33 ± 1.12	28.13 ± 7.37	<0.001
**MCHC (**g/dL)	111	32.54 ± 1.83	115	33.90 ± 1.43	<0.001	109	31.84 ± 1.24	33.1 ± 8.97	<0.001	114	31.97 ± 1.07	32.53 ± 8.19	0.011
**RDW (**cv%)	108	15.89 ± 1.59	107	13.3 ± 1.73	<0.001	109	15.58 ± 1.46	13.62 ± 4.16	<0.001	113	15.51 ± 1.27	13.77 ± 3.66	<0.001

SD: Standard Deviation; Hb: Haemoglobin; **MCV**: Mean corpuscular volume; **MCH**: Mean corpuscular haemoglobin; **MCHC**: Mean corpuscular haemoglobin concentration; **RDW**: Red cell distribution width.

**Table 4:** Pregnancy complications of cases and controls.

	Cases^*^	Controls^*^	OR (95% CI)	P value
	(N = 120)	(N = 120)		
**Gestational diabetes**	5(4.2)	6(5)	0.83 [0.25, 2.78]	0.758
**Pregnancy-induced hypertension**	4(3.3)	5(4.2)	0.79 [0.21, 3.03]	0.734
**Preeclampsia**	0	0	–	0.316
**Eclampsia**	0	0	–	0.316
**IUGR**	4(3.3)	4(3.3)	1.00 [0.24, 4.09]	0.605
**Polyhydramnios**	0	1(0.8)	–	0.368
**Oligohydramnios**	4(3.3)	3(2.5)	1.34 [0.29, 6.14]	0.560
**Placental abruption**	0	0	–	0.316
**Preterm labor**	1(0.8)	2(1.7)	0.5 [0.04, 5.54]	0.311

**IUGR**: Intrauterine growth restriction

* Data expressed as n (%) unless otherwise indicated.

LSCS was the main mode of delivery in cases (61%), while the NVD was the main mode of delivery in controls (55%) (Table 5). There were significant differences (<0.05) in modes of delivery between cases and controls. None of the perinatal and neonatal outcomes were significantly different between cases and controls.

**Table 5:** Labor, perinatal and neonatal outcomes of cases and controls.

	Cases^*^ (N = 111)	Controls^*^ (N = 113)	OR (95% CI)	P value
**Cesarean delivery**	68 (61%)	50 (45%)	1.99 [1.17, 3.39]	0.034^a^
**Postpartum haemorrhage**	2(1.7)	2(1.7)	1.02 [0.14, 7.36]	0.875
**Packed cell transfusion**	2(1.7)	2(1.7)	1.02 [0.14, 7.36]	0.875
**Apgar score at 5 min (<7)**	2(1.7)	2(1.7)	1.02 [0.14, 7.36]	0.875
**Resuscitation at birth**	0	0	–	0.605
**Liquor**	0	0	–	0.605
**Birth weight**				0.222
Male	2.89 ± 0.39	2.96 ± 0.45	−0.17 [−0.43, 0.1]	
Female	2.81 ± 0.34	2.84 ± 0.34	−0.09 [−0.35, 0.17]	
**Body length**				0.253
Male	49.43 ± 2.41	49.53 ± 2.93	−0.04 [−0.3, 0.22]	
Female	49.04 ± 1.94	49.05 ± 2.63	0 [−0.27, 0.26]	
**Head circumferences**				0.771
Male	34.10 ± 1.37	34.10 ± 1.61	0 [−0.26, 0.26]	
Female	33.66 ± 1051	33.75 ± 1.38	−0.06 [−0.32, 0.2]	
**Neonatal ICU admission**	2(1.7)	3(2.5)	0.67 [0.11, 4.11]	0.797
**Congenital abnormalities**				0.519
Hole in the heart	1(0.8)	0	–	
Cleft lip	0	1(0.8)	–	
No abnormalities	110(91.7)	112(93.3)	1.00[0.69, 1.45]	
**Neonatal complications**				0.519
Heart murmurs	1(0.8)	0	–	
Respiratory distress	0	1(0.8)	–	
No complication	110(91.7)	112(93.3)	1.00[0.69, 1.45]	

* Data expressed as n (%) unless otherwise indicated.

^a^Adjusted P value = 0.195.

## Discussion

The present study compared 120 pregnant mothers with beta thalassemia trait (cases) with 120 pregnant mothers without beta thalassemia trait (controls) for pregnancy outcomes. No statistically significant differences were identified between those with trait and those without regarding age, BMI, ethnicity, district of residence, occupation and monthly income.

Most participants in both groups did not develop any noteworthy symptoms during the three trimesters. However, a higher number were reported for each symptom from traits than from controls. The most frequently reported symptom in the 1^st^ trimester was morning sickness (40% in cases & 37.5% in controls; p = 0.462), but as expected the symptom declined gradually through the trimesters. The number of participants with tiredness increased in the 3^rd^ trimester (31.7% in case & 19.2% p = 0.066 in controls). The reported incidence of headache was different between the two groups. Both in the 2^nd^ (13.3% to 7.5%) and 3^rd^ trimester (17.5% vs 5.8%) more cases than controls were affected with the headache whereas a statistically significant difference (unadjusted P = 0.011) was observed initially in 3^rd^ trimester. However, It was no longer significant after correction for multiple comparisons (adjusted P = 0.168).

Thalassaemia traits would be expected to be more anaemic than the controls to begin with. In the current study, almost all tested full blood count parameters (Hb, MCV, MCH, MCHC, and RDW) showed a significant difference between the two groups (Table 3). Both cases and controls showed a drop in hemoglobin levels in the 2^nd^ trimester, only to rise in the 3^rd^ trimester, to reach values similar to those in the 1^st^ trimester. These findings are similar to those of the previous studies by Tabrizi and Barjasteh (2015) and Akinbami *et al.* (2013) [9,10]. A low prenatal hemoglobin status of the mother can cause low birth weight in neonates [9]. Careful monitoring of anaemic beta thalassaemia trait women for further worsening of anemia and the development of pregnancy-associated complications is therefore rational.

It may seem an instinctive practice to transfuse blood for pregnant mothers when their haemoglobin levels decrease to minimize maternal or fetal complications. Defining when to transfuse in pregnancy, especially in situations where the mother has a hereditary anaemia seems loosely defined. There was a statistically significant increase in blood transfusions between cases and controls in previous pregnancies and during the current pregnancy (adjusted P < 0.001). Among the cases, 22 (18.3%) and 20 (16.7%) had undergone a blood transfusion in 2^nd^ and 3^rd^ trimester, respectively. Only one participant from the control group underwent a blood transfusion in 3^rd^ trimester and none during the 2^nd^ trimester. A similar finding has been reported by Sheiner *et al*. (2024) [11]. The haemoglobin level which induced the decision for blood transfusion ranged from 6.7–7.4 g/dl with a mean of 7.1 ± 0.23 g/dL in the 2^nd^ trimester while the mean value was 7.7 ± 0.23 g/dL in the 3^rd^ trimester. When analyzing the changes of Hb levels during the three trimesters in 10 random beta thalassaemia trait mothers who had undergone blood transfusions (Fig 1) all of them had started their pregnancy with disproportionately low Hb levels and showed a further decrease in Hb levels at the end of the second trimester, followed by a proportionate increase in the third trimester. None of the mothers had excessive symptoms nor did they demonstrate fetal insufficiency despite the low haemoglobin levels. It is clear that the decision to transfuse blood was purely made due to the low haemoglobin value as the sole criterion.

The most frequent medical complication of pregnancy was gestational diabetes found in 5/120 (4.2%) in cases and 6/120 (6%) in controls (p = 0.758). This was followed by pregnancy-induced hypertension 4 (3.3%) and 5 (4.2%) in cases and controls, respectively (P = 0.734).

IUGR (3.3% in each group), oligohydramnios (3.3% and 2.5% in cases and controls) and pre-term labor (0.8% and 1.7% in cases and controls) were found in a small number of individuals in either group. None of the participants developed preeclampsia, eclampsia and placental abruption. Importantly none of the complications were significantly different between cases and controls. This contrasts with a study by Amoee *et al.* (2011) and Sheiner *et al.* (2004) [7,11] which found a higher prevalence of oligohydramnios among thalassaemia traits, while, Vafaei *et al.* (2020) [12] and Charoenboon (2015) [13] failed to find such a high prevalence. Ruangvutilert *et al.* (2022) reported an increase in pregnancy-induced hypertension among thalassaemia traits [14]. Charoenboon (2015) found a statistically significant increase in pre-term labor among beta thalassaemia traits [13].

Several studies that investigated pregnancy outcomes of beta thalassemic traits found higher rates of cesarean delivery [12]. The cesarean delivery rate in our study among thalassaemia traits was 61% compared to the controls (45%) (p = 0.034; adjusted P = 0.195). We were unable to identify an exact indication for the cesarean delivery in most of these patients. According to the Royal College of Obstetricians and Gynecologists, thalassaemia trait in itself is not an indication of a cesarean section and the delivery can be planned according to local guidelines in uncomplicated pregnancies [15]. In Sri Lanka, there are no firm guidelines laid down on criteria for the choice of delivery in uncomplicated pregnancies. A recent study had identified that LSCS rate is as high as 40% in the country [16]. Caesarean deliveries are not limited to pregnant mothers who have obstetric complications. Therefore, the decision on the mode of delivery is influenced by the personal views of the clinician and also the patient’s preference.

In the current study, none of the perinatal outcomes, including postpartum hemorrhage, packed cell transfusion, and Apgar score at 5 min and neonatal ICU admissions were significantly different. Our results show no significant difference between cases and controls in congenital abnormalities and neonatal complications either. A neonate’s birth weight can depend on several parameters, including gestational age, sex, distance from a previous pregnancy, maternal weight, type of delivery, number of abortions, and birth rank [17]. In the control group, the mean body weight of males was 2.97 kg and of females was 2.85 kg, whereas in the cases group, the mean body weight of males and females were 2.89 kg and 2.81 kg, respectively (p = 0.22). These values are comparable to the average birth weight of boys and girls from the western province of Sri Lanka where of 2215 neonates born in the Gampaha district (in the Western Province), Sri Lanka, the mean birth weights of boys and girls were 2.97 kg and 2.8 kg, respectively [18]. The number of cases of low birth weight in cases (n = 19/111; 17.12%) was also not significantly different from that of the controls (n = 17/113; 15.04%) (p = 0.80).

We acknowledge several limitations in our study. The diagnosis of beta thalassemia trait was based solely on the HPLC quantitative method, without genetic confirmation, which is a technical limitation. Additionally, the specific type of beta thalassemia mutation, the possible coexistence of a triple alpha gene condition, and iron deficiency, some factors that may influence hemoglobin levels in pregnant women, were not assessed in this study. Furthermore, the study population was drawn primarily from a single thalassemia center, which may limit the generalizability of the findings due to regional variations in geography, lifestyle, and dietary practices. To enhance the robustness of future research, studies should consider increasing the sample size, including participants from multiple centers and diverse geographic locations, and incorporating long-term maternal and fetal follow-up.

## Conclusions

The results of our study suggest that during pregnancy most pregnant mothers with thalassaemia trait do not experience symptoms in any of the three trimesters: but of those who experience symptoms headache seems to be proportionately more than in those without trait. Individuals with beta thalassaemia trait are transfused more blood during the pregnancy, especially during the second and third trimesters; a judgment perhaps induced by lower Hb levels of the mother rather than any maternal or fetal complications. We were unable to show high levels of maternal or fetal complications in beta thalassaemia except for high rates of caesarean section. The latter we believe had nothing to do with the thalassaemia pregnancy but merely reflected the obstetrician’s choice.
